# Supplementary material for: Screening of homing and tissue-penetrating peptides by microdialysis and in vivo phage display
Source: Life Sci Alliance. 2025 Feb 11;8(5):e202201490. doi: 10.26508/lsa.202201490 (PMC11814485; doi:10.26508/lsa.202201490)
Supplement: Supplementary file 1 [file LSA-2022-01490_TableS1.docx]

## Supplementary tables

**Table S1.** The number of rescued phage clones (the C7XC library screen) from skin wound and normal skin microdialysates at different time points.

| Time | Total phage in wound dialysate | Total phage in normal skin dialysate |
| --- | --- | --- |
| 60´ | 810 | 0 |
| 120´ | 1920 | 0 |
| 200´ | 200 | 0 |
| 260´ | 45 | 15 |
| 290´ | 22 | 0 |
