# Supplementary material for: Screening of homing and tissue-penetrating peptides by microdialysis and in vivo phage display
Source: Life Sci Alliance. 2025 Feb 11;8(5):e202201490. doi: 10.26508/lsa.202201490 (PMC11814485; doi:10.26508/lsa.202201490)
Supplement: Supplementary file 2 [file LSA-2022-01490_TableS2.docx]

**Table S2.** Details of the peptide motif enrichment analysis of the high-throughput sequencing data from the skin wound samples and the dialysate samples. The numbers represent the relative number of the motifs compared with the naïve library.

| **Motif** | **CKDQT** | **NSLW** | **YEHH** | **FQxF** | **WHSxC** | **ExKCD** | **WxLIC** | **YxWY** |
| --- | --- | --- | --- | --- | --- | --- | --- | --- |
| **1 motif per number of motifs in naïve library^a^** | absent | 262144 | 262144 | 209715 | 209715 | 149796 | 149796 | 141957 |
| **Total blood^b^** |  | CNSLWYLMC |  |  | CMGVWHSLC |  |  | CNIDYTWYC |
| **Wounds^c^** | CKDQTVYMC | CLNSLWMDC | CIIYYEHHC | CFQSFRARC | CKWIWHSEC | CCQEGKCDC | CNEQWLLIC | CNIDYTWYC |
|  | CKDQTYPKC | CNKNSLWSC | CINYEHHPC | CDYSFQTFC | CWLRWHSFC | CESKCDKEC | CLDDWSLIC | CFYIWYHEC |
|  | CCKDQTKKC | CNSLWYLMC | CSYTYEHHC | CSWDFQLFC | CWTIWHSHC | CCQEGKCDC | CNEIWHLIC | CSKYVWYYC |
|  | CKDQTVYMC |  | CYEHHYGWC | CIFQYFSRC | CNKLWHSTC |  |  | CYTWYNKEC |
|  | CCKDQTKKC |  | CEDYEHHEC | CYMFQLFCC | CSYWWHSEC |  |  | CCYAWYSFC |
|  | CKDQTHLQC |  |  | CFFQWFQFC |  |  |  | CYYWYLLIC |
|  |  |  |  | CDSAFQDFC |  |  |  | CYSWYGQHC |
|  |  |  |  |  |  |  |  | CEYEWYMDC |
|  |  |  |  |  |  |  |  | CIYMWYRDC |
|  |  |  |  |  |  |  |  | CYEWYRDQC |
|  |  |  |  |  |  |  |  | CYCWYSDYC |
|  |  |  |  |  |  |  |  | CYTWYIWKC |
|  |  |  |  |  |  |  |  | CYYRWYYNC |
|  |  |  |  |  |  |  |  | CRYYWWYLC |
|  |  |  |  |  |  |  |  | CYIWYHHLC |
| **Microdialysate^d^** |  |  |  | CEFQTFYNC | CWTIWHSHC | CEWKCDLDC | CDNWLLICC | CYEYMWYYC |
|  |  |  |  | CIFQRFRRC | CSHMWHSIC | CLKELKCDC | CDMFWHLIC | CISYEWYKC |
|  |  |  |  | CDYSFQTFC | CYYEWHSSC | CLEDKCDGC | CNMYWKLIC | CYQWYSKHC |
|  |  |  |  | CFQFFYHDC |  |  | CNYHWNLIC | CYQWYARFC |
|  |  |  |  | CFQKFSQYC |  |  |  |  |

Continues

| **Motif** | **EIYC** | **CYKxW** | **FLY** | **YxWH** | **HYDN** | **FIxL** | **CxxYNY** | **YDxIC** |
| --- | --- | --- | --- | --- | --- | --- | --- | --- |
| **1 motif per number of motifs in naïve library^a^** | 87381 | 87381 | 74898 | 74898 | 87381 | 61681 | 49932 | 49932 |
| **Total blood^b^** | CSKYDEIYC | CYKCWGHNC | CLMFLYSYC | CYDWHDHWC | CDHYDNDIC | CFIELRICC | CSNYNYEIC | CSSDYDYIC |
|  | CCWHDEIYC |  | CFLYESLNC |  |  | CFIQLNQNC |  |  |
|  |  |  | CIFFLYHIC |  |  | CCHFIDLNC |  |  |
|  |  |  | CLMFLYYDC |  |  |  |  |  |
| **Wounds^c^** | CYKSDEIYC | CYKLWYHIC | CIIFLYSC | CPDTYHWHC | CWTLHYDNC | CYFIMLSMC | CYIYNYKEC | CDMCYDWIC |
|  | CIIMHEIYC | CYKNWAMKC | CFLYQHWSC | CYYWHTSTC | CHYDNSTKC | CLFIMLIDC | CHMYNYYDC | CLPQYDKIC |
|  | CSYNSEIYC | CYKNWHNDC | CRMFLYLKC | CFYSWHYTLC | CHYDNSLIC | CLWFICLLC | CYIYNYKEC | CLKNYDLIC |
|  | CSKYDEIYC |  | CFLYWLNAC | CYMWHNIKLC | CHYDNKKLC | CFIYLCKQC | CILYNYIQC | CFMWYDQIC |
|  | CSSMREIYC |  | CSNFLYQFC | CGYMYDWHC | CWDYHYDNC | CSMFFIHLC | CCNNYNYHC | CWCFYDHIC |
|  | CLKCEEIYC |  | CMSFLYQC | CYLWHMNYC | CMLEHYDNC | CFIDLICIC | CWIYNYSIC | CMAYDMIC |
|  | CYYLLEIYC |  | CYFLYLKLC | CSDYKWHYC |  | CFIWLEEIC | CYIYNYKEC | CAYCKYDNIC |
|  | CQLTLEIYC |  | CFLYYKTLC | CSYNWHYLC |  |  | CRFYNYMHC | CEMTYDLIC |
|  | CNKNKEIYC |  | CFLYSRDHC | CSYWWHSEC |  |  | CSNYNYEIC | CWSPYDEIC |
|  | CSKYDEIYC |  | CLMFLYYDC | CYNWHYHYC |  |  |  |  |
|  | CCNQLEIYC |  | CYDFLYDDC |  |  |  |  |  |
|  | CCENDEIYC |  | CRFLYLMSC |  |  |  |  |  |
|  | CSKWEEIYC |  | CLSHFLYRC |  |  |  |  |  |
|  | CNNHLEIYC |  |  |  |  |  |  |  |
|  | CYKIGEIYC |  |  |  |  |  |  |  |
| **Microdialysate^d^** | CWYNHEIYC | CCYKWWFDC | CCTFLYRYC | CYYWHTIC | CWTLHYDNC | CANFINLPTC | CQGYNYEWC | CYDMICLEC |
|  |  | CYKCWTCYC | CNFLYMSLC | CYYEWHSSC | CHYDNDYVC | CRISFIKLC |  | CRRMYDSIC |
|  |  | CYKVWNQIC | CLMFLYYDC | CYDWHYGKC | CHYDNKKLC | CFIFLAKYC |  | CSSAYDDIC |
|  |  | CYKSWLLKC | CCSFLYLDC |  | CHYDNSTKC | CSHFIILRC |  |  |
|  |  |  |  |  |  | CFIQLKSDC |  |  |
|  |  |  |  |  |  | CKFFICLLC |  |  |

Continues

| **Motif** | **RxCQ** | **NYHC** | **KSxLK** | **LMxQC** | **CIIxxE** | **QCxT** | **FxxMQ** | **ADQ** |
| --- | --- | --- | --- | --- | --- | --- | --- | --- |
| **1 motif per number of motifs in naïve library^a^** | 41943 | 38836 | 36158 | 36158 | 36158 | 36158 | 31775 | 7182 |
| **Total blood^b^** | CRWCQQNCC | CEKQNNYHC | CKMKSNLKC |  |  |  |  |  |
|  | CRMCQFHEC |  |  |  |  |  |  |  |
| **Wounds^c^** | CSIRQCQAC | CPTTKNYHC | CHKSTLKEC | CCSLMKQC | CIIYYEHHC | CHFDQCLTC | CFMEMQQMC | CRYQADQKC |
|  | CMTLRQCQC | CEKQNNYHC | CHKSTLKEC | CRNLLMLQC | CIIMHEIYC | CMEQCMTMC | CFSMMQKEC | CRCSIADQC |
|  | CRACQRDIC | CEKQNNYHC | CDSKSTLKC | CRDRLMHQC | CIIIKEQNC | CDQQCCTLC | CNFNHMQHC | CYMQYADQC |
|  | CRACQRDIC | CPLKTNYHC | CDKSILKNC | CHGILMHQC | CIIGYEIIC | CMIQCYTYC | CSMFDCMQC | CESLHADQC |
|  | CRSCQFDKC | CKTLNNYHC | CNKSGLKTC | CIKELMYQC | CIITKESMC | CDWYQCMTC | CCFDMMQEIC | CADQLIQAC |
|  | CRACQRDIC | CETNDNYHC | CHKSGLKPC |  |  | CQCDTYKHC | CFHDMQDDC | CADQDKMFC |
|  | CMRICQRMC | CFFHYNYHC | CYKSHLKKC |  |  | CKQCMTEMC | CHFCFMQLC |  |
|  | CQRACQSNC | CCNNYNYHC | CNNKSGLKC |  |  | CIYCQCYTC | CFNHMQTDC |  |
|  | CRDCQKLEC | CMIWQNYHC | CNKSLLKPC |  |  | CRYIQCITC | CRNFMHMQC |  |
|  | CRACQRDIC | CLLCTNYHC | CKSTLKPQC |  |  | CTFHQCDTC | CFHDMQDDC |  |
|  | CETWRHCQNEC | CFDQCNYHC | CDKSILKNC |  |  | CFNWQCETC | CRFYYMQQC |  |
|  |  | CDWSYNYHC |  |  |  | CCQCQTLHC | CFAWMQWQC |  |
|  |  | CYTDVNYHC |  |  |  | CQQCYTHWC |  |  |
|  |  |  |  |  |  | CYQCKTKSTC |  |  |
|  |  |  |  |  |  | CQSQCTTCDC |  |  |
|  |  |  |  |  |  | CQCITYVHC |  |  |
|  |  |  |  |  |  | CQCCTNDYC |  |  |
| **Microdialysate^d^** | CYIHRFCQC | CFDQCNYHC | CYKSILKKC | CFCQLMYQC |  | CMIQCYTYC | CFSLMQLLC | CADQLIQAC |
|  | CQDRNCQWC |  | CKSMLKILC | CHGILMHQC |  |  |  | CADQWFNKC |
|  | CARQCQSKC |  | CQKKSWLKC | CCDILMIQC |  |  |  | CFKHADQTDC |
|  | CRYLRACQC |  | CLKSNLKNC | CFCQLMYQC |  |  |  | CWWFHADQC |
|  |  |  |  |  |  |  |  | CDMKQADQC |

Continues

| **Motif** | **LxCQ** | **EINxC** | **QSxW** | **KVxM** | **CHYxN** | **QCxQ** | **QLxW** |  |
| --- | --- | --- | --- | --- | --- | --- | --- | --- |
| **1 motif per number of motifs in naïve library^a^** | 28340 | 25575 | 25575 | 23302 | 22310 | 21399 | 20560 |  |
| **Total blood^b^** | CLLSCQYNC | CHFNEINTC | CYQSEWDMC | CKVNMGSIC |  | CFDQCWQC | CYRSQLSWC |  |
|  | CKLNCQDEC |  | CQSDWYC | CHKVYMMNC |  | CEQCWQMC | CPQQLDWIC |  |
|  |  |  |  |  |  | CQIQCQQSC |  |  |
| **Wounds^c^** | CLDCQNELC | CNIYEINSC | CSQSSWFLC | CKNKVDMMC | CHYDNSTKC | CQCCQMYQC | CNQLSWKQC | CNIPQLHWC |
|  | CEVLYCQWC | CHFNEINTC | CQSDWRFWC | CKVIMCLEC | CHYDNSLIC | CCQWTQCQQYC | CWQLTWVKC | CQLTWFQNC |
|  | CDLMCQDNC | CKKNEINNC | CETDYQSEWC | CWNKVNMSC | CYCHYINC | CQCYQFHQC | CWNCQLDWC | CSQLKWTFC |
|  | CWIELLCQC |  | CQRQSIWSC | CNKKVKMNC | CHYKNISYC | CQFEQCYQC | CQLKWKQSC | CWNCQLDWC |
|  | CQCQLHCQC |  | CQSQWCCSC | CDKVNMDTC | CHYYNDVIC | CQCDQYSWC | CFQLKWHMC | CHQLMWLLC |
|  | CLCLRCQWC |  | CMQSQWHMC |  | CHYHNLIYC | CFIMQCHQC | CQLKWMITC | CLGSQLSWC |
|  | CEPYLLCQC |  | CADMQSYWC |  | CHYDNKKLC | CQCSQNMSC | CQQLDWMNC | CLDMQLHWC |
|  | CLCKLQCQC |  | CEQSHWKQC |  | CHYMNYC | CWNIQCWQC | CHQLMWMLC | CLDSQLSWC |
|  | CLDCQNELC |  | CMQSNWEQC |  | CHYENCGIC | CQCLQYYNC | CHLQLIWYC |  |
|  | CLNCQLEIC |  | CKYQSQWIC |  | CHYNNWMLC | CFLQCSQSC | CQLCWSQHC |  |
|  | CFLSCQCCC |  | CQSHWKNC |  | CHYHNLKLC | CSQCSQCSC | CQLHWNTEC |  |
|  | CMLWCQDQC |  |  |  | CHYTNIEHC | CWNIQCWQC | CQLRWNYSC |  |
|  | CLWCQKNCC |  |  |  |  | CQCAQTWC | CQLKWNDTC |  |
|  | CSLSCQLNC |  |  |  |  | CFFQCYQCC | CLDMQLHWC |  |
|  | CHNMLQCQC |  |  |  |  | CLLWQCDQC | CQLQWQQNC |  |
|  | CDKLLKCQC |  |  |  |  |  | CWSQLEWC |  |
|  |  |  |  |  |  |  | CQLQWDKYC |  |
|  |  |  |  |  |  |  | CSSNQLIWC |  |
| **Microdialysate^d^** | CLDCQNELC | CKKNEINNC | CQSDWHWMC | CYSKVDMNC | CHYQNYQQC | CFDQCWQC |  |  |
|  |  | CQYKEINKC | CMHQQSEWC | CDWKVPMGC | CHYDNDYVC | CQCCQDWWC |  |  |
|  |  | CKKNEINNC | CQSMWYLQC | CNKKVKMNC | CHYDNKKLC | CDQCEQSNSKC |  |  |
|  |  | CDRHEINAC | CADMQSYWC | CLNKVKMIC | CHYDNSTKC | CQQCLQC |  |  |
|  |  |  | CGEHQSKWC |  |  |  |  |  |
|  |  |  | CQSQWCCSC |  |  |  |  |  |

Continues

| **Motif** | **HSxKC** | **KQxEC** | **DIV** | **TQxKC** | **YxYD** | **HHxT** | **WxES** | **NLxP** |
| --- | --- | --- | --- | --- | --- | --- | --- | --- |
| **1 motif per number of motifs in naïve library^a^** | 18725 | 18725 | 17190 | 16644 | 13981 | 13981 | 10082 | 9892 |
| **Total blood^b^** | CWDAHSNKC |  |  | CYNSTQNKC | CYNYDDPNC | CSHNHHTTC | CYWQESEKC |  |
|  |  |  |  |  | CKYKYDYQC | CHHSTQSYC | CTWVESMIC |  |
|  |  |  |  |  | CDCYDYDQC | CYNEHHSTC |  |  |
| **Wounds^c^** | CWDAHSNKC | CGSKQGECC | CSSDIVDEC | CWMDTQAKC | CYQYDIC | CNEMHHVTC | CRWWESSCC | CLCCNLLPC |
|  | CLKDHSHKC | CDKQNECKC | CLGWDIVNC | CPKHTQKKC | CWSFYSYDC | CCNHHNTWC | CWLESDMDC | CNTNLKPKC |
|  | CSMYHSEKC |  | CDEDIVSCC | CDEKTQSKC | CHWLYDYDC | CSYHHCTIC | CWTESRMKC | CHNLQPKSC |
|  | CSKSHSHKC |  | CYDIVNHCC | CNKHTQHKC | CKNYLYDYC | CYKNHHSTC | CSTCWDESC | CNLHPVQNC |
|  | CLYHSKKC |  | CNYDIVGKC | CDTETQKKC | CHWEYWYDC | CYKNHHSTC | CSLWWESLC | CNLLPEKTC |
|  | CLHIHSNKC |  | CQKVDIVEC |  | CFKYDYDYC | CSHHQTHDC | CWYESSMQC | CLVVNLKPC |
|  |  |  | CHGCIDIVC |  | CYYYDTENC | CHLHHMTCC | CSLWWESLC | CLNLTPKNC |
|  |  |  |  |  | CSWYAYDIC | CSFNHHFTC | CYNWEESC | CNLWPICTC |
|  |  |  |  |  | CYEYDLEQC | CLHHKTLSC | CYWMESMKC | CNKNLNPCC |
|  |  |  |  |  | CGYMYDWHC | CLHHSTNQC | CNWIWLESC | CMGNLIPWC |
|  |  |  |  |  | CSQYYYDQC |  | CILWWESHC | CMTENLTPC |
|  |  |  |  |  | CYMYDYYYC |  | CWVESCHEC |  |
|  |  |  |  |  | CMSYKYDLC |  | CYWNESYSC |  |
|  |  |  |  |  | CYPYDLRMHC |  | CWYESDQC |  |
|  |  |  |  |  | CWDYHYDNC |  | CRHWRESDC |  |
|  |  |  |  |  | CLIYYYYDC |  | CWMESNRWC |  |
|  |  |  |  |  | CCFYDYDDC |  | CSWMESKNC |  |
| **Microdialysate^d^** | CSIVHSKKC | CDSLKQTEC | CDIVSTLHC | CYEHTQNKC | CLEYFYDCC | CSYHHCTIC | CSWCESSWC | CNNLDPPNC |
|  | CLTMHSCKC | CDEKQNECC | CEDIVMHNC | CQVGTQVKC | CCRDYSYDC | CCKHHQTWC | CWLESDMDC | CYVNLNPMC |
|  | CFLEHSIKC | CFLKQFEC | CDIDIVKNC | CRTATQNKC | CMLLYMYDC | CLHNHHSTC | CSEWHESNC | CLNLLPNLC |
|  | CEDFHSMKC |  |  | CDEKTQSKC | CSWYAYDIC | CHHVTDKLC | CLWWWESNC | CQINNLEPC |
|  |  |  |  |  |  | CHHITTPCC |  |  |

^a^The bigger the number, the rarer the motif. Example: 1/262144 for NSLW.

^b^30 min / 5 h / skins. All samples from blood and all skin samples as controls.

^c^Total five wounds, four from one 30 min experiment and one from 5 h experiment.

^d^Two probes that gave back phage.
