# Supplementary material for: Screening of homing and tissue-penetrating peptides by microdialysis and in vivo phage display
Source: Life Sci Alliance. 2025 Feb 11;8(5):e202201490. doi: 10.26508/lsa.202201490 (PMC11814485; doi:10.26508/lsa.202201490)
Supplement: Supplementary file 3 [file LSA-2022-01490_TableS3.docx]

**Table S3.** Details of BLAST analysis (Altschul et al., 1990) of the peptide sequences (performed without cysteines). The first sequence hits present in the proteome of *Homo sapiens* (hu), *Rattus norvegicus* (rat) and *Mus musculus* (ms) are presented here. The gene names and location information are obtained from the UniProt database (The UniProt Consortium, 2019).

| **CPKKHHLDC** | | | | | | |
| --- | --- | --- | --- | --- | --- | --- |
| **Protein name** | **Gene name** | **Percent identity** | | **PKKHHLD** | **Location (extracellular)** | **Location (intracellular)** |
| Zinc finger protein 142 | ZNF142 | hu | 100 | PKKHHLD |  | Nucleus |
|  |  | rat | 60.00 | --KHYLE |  |  |
|  |  | ms | 60.00 | --KHYLE |  |  |
| Clathrin coat assembly protein AP180 | SNAP91 | hu | 85.71 | PKKKHLD | Cell membrane |  |
|  |  | rat | 85.71 | PKKKHLD |  |  |
|  |  | ms | 85.71 | PKKKHLD |  |  |
| Phosphatidylinositol-binding clathrin assembly protein | PICALM | hu | 85.71 | PKKKHLD | Cell membrane | Cytosol |
|  |  | rat | 85.71 | PKKKHLD |  | Endosome |
|  |  | ms | 85.71 | PKKKHLD |  | Golgi apparatus |
|  |  |  |  |  |  | Nucleus |

| **CYHDTYPNC** | | | | | | |
| --- | --- | --- | --- | --- | --- | --- |
| **Protein name** | **Gene name** | **Percent identity** | | **YHDTYPN** | **Location (extracellular)** | **Location (intracellular)** |
| Teneurin-1 | TENM1 | hu | 85.71 | YHDTYPD | Cell membrane | Cytoskeleton |
|  |  | rat | 85.71 | YHDTYPD | Secreted | Golgi apparatus |
|  |  | ms | 85.71 | YHDTYPD |  | Endoplasmic reticulum |
|  |  |  |  |  |  | Nucleus |
| Leusine-rich repeat-containing protein 7 | LRRC7 | hu | 71.43 | YHDSNPN | Cell membrane | Cytoskeleton |
|  |  | rat | 71.43 | YHDPTPN | Secreted | Cytosol |
|  |  | ms | 71.43 | YHDPTPN |  | Nucleus |
| Discoidin, CUB and LCCL domain-containing protein 2 | DCBLD2 | hu | 71.43 | YPQTYPN | Cell membrane |  |
|  |  | rat | 71.43 | YPGTYPN |  |  |
|  |  | ms | 71.43 | YPGTYPN |  |  |
| Multiple epidermal growth factor-like domains protein 9 | MEGF9 | hu | 71.43 | YHDSIPN | Basement membrane |  |
|  |  | rat | 71.43 | YHDSIPN |  |  |
|  |  | ms | 71.43 | YHDSIPN |  |  |
| Soluble calcium-activated nucleotidase 1 | CANT1 | hu | 83.33 | YNDTYP | Cell membrane | Endoplasmic reticulum |
|  |  | rat | 83.33 | YNDTYP | Secreted | Golgi apparatus |
|  |  | ms | 83.33 | YNDTYP |  |  |
| Coatomer subunit zeta-1 | COPZ1 | hu | 83.33 | YDDTYP |  | Golgi apparatus |
|  |  | rat | 83.33 | YDDTYP |  |  |
|  |  | ms | 83.33 | YDDTYP |  |  |

| **CNYLVEKNC** | | | | | | |
| --- | --- | --- | --- | --- | --- | --- |
| **Protein name** | **Gene name** | **Percent identity** | | **NYLVEKN** | **Location (extracellular)** | **Location (intracellular)** |
| R3H domain-containing protein 1 | R3HDM1 | hu | 70.00 | NYGKILVEKN |  |  |
|  |  | rat | 50.00 | NY---IIDK- |  |  |
|  |  | ms | 50.00 | NY---IIDK- |  |  |
| Titin | TTN | hu | 100 | NYLVDK | Secreted | Cytoskeleton |
|  |  |  |  | NYIVEK |  | Cytosol |
|  |  |  |  | NYVVEK |  | Nucleus |
|  |  |  |  | NYIIEK |  |  |
|  |  |  |  | NYVIEK |  |  |
|  |  |  |  | -YLVE |  |  |
|  |  |  |  | NYIVE |  |  |
|  |  |  |  | NYVVE |  |  |
|  |  |  |  | NYILEK |  |  |
|  |  |  |  | -YLLEK |  |  |
|  |  |  |  | NYHIEK |  |  |
|  |  |  |  | --LVEK |  |  |
|  |  |  |  | -YMVE |  |  |
|  |  |  |  | NYTLEK |  |  |
|  |  |  |  | NYYLEK |  |  |
|  |  |  |  | --LVMKN |  |  |
|  |  | rat | 83.33 | DYSIEK |  |  |
|  |  | ms | 83.33 | NYIVEK |  |  |
|  |  |  |  | NYLVKD |  |  |
|  |  |  |  | NYIVEK |  |  |
|  |  |  |  | -YLVEK |  |  |
|  |  |  |  | NVVEK |  |  |
|  |  |  |  | NYIIEK |  |  |
|  |  |  |  | DYLVE |  |  |
|  |  |  |  | NYVIEK |  |  |
|  |  |  |  | -YLVE |  |  |
|  |  |  |  | NYIVE |  |  |
|  |  |  |  | NYVVE |  |  |
|  |  |  |  | NYILEK |  |  |
|  |  |  |  | NYHIEK |  |  |
|  |  |  |  | -YMVE |  |  |
|  |  |  |  | NYTLEK |  |  |
|  |  |  |  | --LVEKN |  |  |
|  |  |  |  | DYSIEK |  |  |
| E3 ubiquitin-protein ligase LNX | LNX | hu | 71.43 | NFLVEKD |  | Cytosol |
|  |  | rat | 71.43 | NFLVEKD |  |  |
|  |  | ms | 71.43 | NFLVEKD |  |  |
| Unconventional myosin-X | MYO10 | hu | 71.43 | DYLLEKN | Cell membrane | Cytoskeleton |
|  |  | rat | 71.43 | DYLLEKN |  | Cytosol |
|  |  |  |  | NYANEK |  | Nucleus |
|  |  | ms | 71.43 | DYLLEKN |  |  |
|  |  |  |  | NYANEK |  |  |
| Protein IMPACT | IMPACT | hu | 85.71 | NILVEKN |  | Cytosol |
|  |  | rat | 85.71 | NILVEKN |  |  |
|  |  | ms | 85.71 | NILVEKN |  |  |
| Putative malate dehydrogenase 1B | MDH1B | hu | 83.33 | YLIEKN | Cell membrane |  |
|  |  | rat | 83.33 | YLIEKN |  |  |
|  |  | ms | 83.33 | YLIEKN |  |  |
| Potassium voltage-gated channel subfamily KQT member 5 | KCNQ5 | hu | 83.33 | YLVEKD | Cell membrane |  |
|  |  | rat | 83.33 | YLVEKD |  |  |
|  |  | ms | 83.33 | YLVEKD |  |  |
| Potassium voltage-gated channel subfamily KQT member 3 | KCNQ3 | hu | 83.33 | YLVEKD | Cell membrane |  |
|  |  | rat | 83.33 | YLVEKD |  |  |
|  |  | ms | 83.33 | YLVEKD |  |  |
| Histone-lysine N-methyltransferase SUV39H1 | SUV39H1 | hu | 83.33 | NYLVQK |  | Nucleus |
|  |  | rat | 83.33 | NYLVQK |  |  |
|  |  | ms | 83.33 | NYLVQK |  |  |
| Protein fem-1 homolog B | FEM1B | hu | 83.33 | YLVENN |  | Cytosol |
|  |  | rat | 83.33 | YLVENN |  | Nucleus |
|  |  | ms | 83.33 | YLVENN |  |  |
| Interleukin-1 receptor-associated kinase 1-binding protein 1 | IRAK1BP1 | hu | 83.33 | NFLVEK |  | Cytosol |
|  |  | rat | 83.33 | NFLVEK |  | Nucleus |
|  |  | ms | 83.33 | NFLVEK |  |  |
| L-amino-acid oxidase | IL4I1 | hu | 83.33 | NYVVEK | Secreted | Lysosome |
|  |  | rat | 83.33 | NYVVEK |  |  |
|  |  | ms | 83.33 | NYVVEK |  |  |

Continues

| **CDDYQQISC** | | | | | | |
| --- | --- | --- | --- | --- | --- | --- |
| **Protein name** | **Gene name** | **Percent identity** | | **DDYQQIS** | **Location (extracellular)** | **Location (intracellular)** |
| Cullin-9 | CUL9 | hu | 71.43 | DDYEEIS |  | Cytosol |
|  |  | rat | 71.43 | DDYEEIS |  |  |
|  |  | ms | 71.43 | DDYEEIS |  |  |
| Cullin-7 | CUL7 | hu | 71.43 | DDYEEIS |  | Cytoskeleton |
|  |  | rat | 50.00 | -DFEEIS |  | Cytosol |
|  |  | ms | 40.00 | --YEEIA |  | Golgi apparatus |
|  |  |  |  |  |  | Nucleus |
| Receptor-type tyrosine-protein phosphatase H | PTPRH | hu | 71.43 | DEYQQLS | Cell membrane |  |
|  |  | rat | 42.85 | EEYQQLA |  |  |
|  |  | ms | 42.85 | EEYQQLA |  |  |
| Palmitoyltransferase ZDHHC18 | ZDHHC18 | hu | 83.33 | EYQQIS |  | Endoplasmic reticulum |
|  |  | rat | 83.33 | EYQQIS |  | Golgi apparatus |
|  |  | ms | 83.33 | EYQQIS |  |  |
| Cadherin-23 | CDH23 | hu | 83.33 | DYEQIS | Cell membrane |  |
|  |  | rat | 60.00 | -YEQIP |  |  |
|  |  | ms | 60.00 | -YEQIP |  |  |
| Slit homolog 2 protein | SLIT2 | hu | 83.33 | DYNQIS | Secreted |  |
|  |  | rat | 83.33 | DYNQIS |  |  |
|  |  | ms | 83.33 | DYNQIS |  |  |

Continues

| **CLSQTYRIC** | | | | | | |
| --- | --- | --- | --- | --- | --- | --- |
| **Protein name** | **Gene name** | **Percent identity** | | **LSQTYRI** | **Location (extracellular)** | **Location (intracellular)** |
| General transcription factor 3C polypeptide 1 | GTF3C1 | hu | 87.50 | LSQTYYRI |  | Nucleus |
|  |  | rat | 57.14 | LTQT-YEL |  |  |
|  |  | ms | 50.00 | -TQT-YEL |  |  |
| MORC family CW-type zinc finger protein 3 | MORC3 | hu | 100 | LSQTY |  | Nucleus |
|  |  |  |  | LSKTVRI |  |  |
|  |  | rat | 100 | LSQTY |  |  |
|  |  | ms | 100 | LSQTY |  |  |
| Alanine--glyoxylate amniotransferase 2, mitochondrial | AGXT2 | hu | 83.33 | SQTFRI |  | Mitochondrion |
|  |  | rat | 83.33 | SQTFRI |  |  |
|  |  | ms | 83.33 | SQTFRI |  |  |
| Neurexophilin-1 precursor | NXPH1 | hu | 83.33 | LSQTFR | Secreted |  |
|  |  | rat | 83.33 | LSQTFR |  |  |
|  |  | ms | 83.33 | LSQTFR |  |  |
| Protocadherin alpha-C1 | PCDHAC1 | hu | 100 | LSQTY-- |  |  |
|  |  | rat | 50.00 | -SSLFRI |  |  |
|  |  | ms | 100 | LSQTY-- |  |  |

Continues

| **CKMLYEYHC** | | | | | | |
| --- | --- | --- | --- | --- | --- | --- |
| **Protein name** | **Gene name** | **Percent identity** | | **KMLYEYH** | **Location (extracellular)** | **Location (intracellular)** |
| Oxidative stress-induced growth inhibitor 1 | OSGIN1 | hu | 87.50 | KMLYPEYH |  |  |
|  |  | rat | 87.50 | KMLYPEYH |  |  |
|  |  | ms | 87.50 | KMLYPEYH |  |  |
| Ribosomal oxygenase 2 | RIOX2 | hu | 71.43 | KMVYIYH |  | Cytosol |
|  |  | rat | 57.14 | KVVYIYH |  | Nucleus |
|  |  | ms | 57.14 | KVVYIYH |  |  |
| Interferon-related developmental regulator 1 | IFRD1 | hu | 83.33 | KMLYEF |  | Nucleus |
|  |  | rat | 66.67 | KVLYEF |  |  |
|  |  | ms | 66.67 | KVLYEF |  |  |
| XK-related protein 4 | XKR4 | hu | 83.33 | KMVYEY | Cell membrane |  |
|  |  | rat | 83.33 | KMVYEY |  |  |
|  |  | ms | 83.33 | KMVYEY |  |  |
| Protein FAM91A1 | FAM91A1 | hu | 83.33 | MLYPYH |  | Golgi apparatus |
|  |  |  |  | -LYIYH |  |  |
|  |  | rat | 83.33 | MLYPYH |  |  |
|  |  |  |  | -LYIYH |  |  |
|  |  | ms | 83.33 | MLYPYH |  |  |
|  |  |  |  | -LYIYH |  |  |
| Centrosomal protein kizuna | KIZ | hu | 83.33 | KKLYEY- |  | Cytoskeleton |
|  |  | rat | 66.67 | -KLYEYN |  |  |
|  |  | ms | 66.67 | -KLYEYN |  |  |
| 28S ribosomal protein S14, mitochondrial | MRPS14 | hu | 83.33 | KMAYEY |  | Mitochondrion |
|  |  | rat | 83.33 | KMAYEY |  |  |
|  |  | ms | 83.33 | KMAYEY |  |  |

| **CLLIYNWSC** | | | | | | |
| --- | --- | --- | --- | --- | --- | --- |
| **Protein name** | **Gene name** | **Percent identity** | | **LLIYNWS** | **Location (extracellular)** | **Location (intracellular)** |
| Bifunctional apoptosis regulator isoform 1 | BFAR | hu | 71.43 | LLVYHWS | Cell membrane | Endoplasmic reticulum |
|  |  | rat | 66.67 | LLVYHW- |  |  |
|  |  | ms | 66.67 | LLVYHW- |  |  |
| T-cell surface glycoprotein CD3 epsilon chain | CD3E | hu | 71.43 | LLVYYWS | Cell membrane |  |
|  |  | rat | 42.85 | MVVYYWS |  |  |
|  |  | ms | 57.14 | MVIYYWS |  |  |
| KICSTOR complex protein SZT2 | SZT2 | hu | 83.33 | LLTYNW |  | Lysosome |
|  |  | rat | 83.33 | LLTYNW |  | Peroxisome |
|  |  | ms | 83.33 | LLTYNW |  |  |

| **CKKNEINNC** | | | | | | |
| --- | --- | --- | --- | --- | --- | --- |
| **Protein name** | **Gene name** | **Percent identity** | | **KKNEINN** | **Location (extracellular)** | **Location (intracellular)** |
| Coiled-coil domain-containing protein 110 | CCDC110 | hu | 85.71 | KKNEKNN |  | Cytoskeleton |
|  |  | rat | 60.00 | --NELHN |  | Nucleus |
|  |  | ms | 83.33 | KKNEKN- |  |  |
| Jouberin | AHI1 | hu | 100 | KNEINN | Cellular junction | Cytoskeleton |
|  |  | rat | 100 | KNEIN- |  | Cytosol |
|  |  | ms | 100 | KNEIN- |  |  |
| Spectrin beta chain nin-erythrocytic 1 | SPTBN1 | hu | 83.33 | KNEIDN | Cell membrane | Cytoskeleton |
|  |  | rat | 83.33 | KNEIDN |  | Cytosol |
|  |  | ms | 83.23 | KNEIDN |  | Nucleus |

| **CLTVLSEQC** | | | | | | |
| --- | --- | --- | --- | --- | --- | --- |
| **Protein name** | **Gene name** | **Percent identity** | | **LTVLSEQ** | **Location (extracellular)** | **Location (intracellular)** |
| Exportin-T | XPOT | hu | 85.71 | LTVLSDQ |  | Cytosol |
|  |  | rat | 71.43 | LSVLSDQ |  | Nucleus |
|  |  | ms | 71.43 | LPVLSDQ |  |  |
| Spectrin alpha chain non-erythrocytic 1 | SPTAN1 | hu | 85.71 | LTVLSEE |  | Cytoskeleton |
|  |  | rat | 57.14 | LSILSEE |  | Cytosol |
|  |  | ms | 57.14 | LSILSEE |  | Nucleus |
| Phospatidylinositol 4-phosphate 3-kinase C2 domain-containing subunit beta | PIK3C2B | hu | 85.71 | LSVLSEQ | Cell membrane | Cytosol |
|  |  | rat | 85.71 | LSVLSEQ |  | Endoplasmic reticulum |
|  |  | ms | 85.71 | LSVLSEQ |  | Nucleus |
| Rho GDP-dissociation inhibitor 3 | ARHGDIG | hu | 85.71 | LTLLSEQ |  | Cytosol |
|  |  | rat | 57.14 | LTLLTEH |  |  |
|  |  | ms | 71.43 | LTLLTEQ |  |  |
| Midasin | MDN1 | hu | 85.71 | ---LSEQ |  | Cytosol |
|  |  |  |  | --VLSE- |  | Nucleus |
|  |  |  |  | --VLIEQ |  |  |
|  |  | rat | 71.43 | LTTLTEQ |  |  |
|  |  | ms | 71.43 | LTTLTEQ |  |  |
| Protein-associating with the carboxyl-terminal domain of ezrin | SCYL3 | hu | 71.43 | LTILNEQ |  | Cytosol |
|  |  | rat | 42.85 | LPIFNEQ |  | Golgi apparatus |
|  |  | ms | 42.85 | LPIFNEQ |  | Lamellipodium |
| TRIO and F-actin-binding protein | TRIOBP | hu | 85.71 | LQVLSEQ |  |  |
|  |  | rat | 71.43 | LQVLSER |  | Cytoskeleton |
|  |  | ms | 71.43 | LQVLSER |  | Nucleus |
| Neurofascin | NFASC | hu | 71.43 | LTVLADQ | Cell membrane |  |
|  |  | rat | 71.43 | LTVLADQ |  |  |
|  |  | ms | 71.43 | LTVLADQ |  |  |
| Delphilin | GRID2IP | hu | 85.71 | LDVLSEQ | Cell membrane |  |
|  |  |  |  | LTQLS-- |  |  |
|  |  | rat | 100 | --VLSEQ |  |  |
|  |  | ms | 71.43 | LDALSEQ |  |  |

Continues

| **CYWEDKNLC** | | | | | | |
| --- | --- | --- | --- | --- | --- | --- |
| **Protein name** | **Gene name** | **Percent identity** | | **YWEDKNL** | **Location (extracellular)** | **Location (intracellular)** |
| Casein kinase I alpha | CSNK1A1 | hu | 85.71 | YREDKNL |  | Cytosol |
|  |  | rat | 85.71 | YREDKNL |  | Cytoskeleton |
|  |  | ms | 85.71 | YREDKNL |  | Nucleus |
| Carboxypeptidase E | CPE | hu | 85.71 | YWEDNKN | Cell membrane | Golgi apparatus |
|  |  | rat | 85.71 | YWEDNKN |  | Nucleus |
|  |  | ms | 85.71 | YWEDNKN |  | Secreted |
| Programmed cell death 1 ligand 1 | CD274 | hu | 75.00 | YWEMEDKN | Cell membrane | Endosome |
|  |  | rat | 62.50 | YWEKEDKE | Secreted |  |
|  |  | ms | 75.00 | YWEKEDEQ |  |  |
| Telomeric repeat binding factor 1 | TERF1 | hu | 85.71 | WEEDKNL |  | Cytoskeleton |
|  |  | rat | 57.14 | WEEDRSL |  | Nucleus |
|  |  | ms | 57.14 | WEEDRIL |  |  |
| Neurite extension and migration factor | NEXMIF | hu | 75.00 | WGEEDKNL |  | Cytosol |
|  |  | rat | 75.00 | WGEEDKNL |  | Cytoskeleton |
|  |  | ms | 75.00 | WGEEDKNL |  | Nucleus |

Continues

| **CFCQLMYQC** | | | | | | |
| --- | --- | --- | --- | --- | --- | --- |
| **Protein name** | **Gene name** | **Percent identity** | | **FCQLMYQ** | **Location (extracellular)** | **Location (intracellular)** |
| Inositol 1,4,5-trisphosphate receptor type 3 | ITPR3 | hu | 80.00 | FCHLM-- | Cell membrane | Cytosol |
|  |  |  |  | ---LMYH |  | Nucleus |
|  |  | rat | 80.00 | FCHLM-- |  |  |
|  |  |  |  | ---LMYH |  |  |
|  |  | ms | 80.00 | FCHLM-- |  |  |
|  |  |  |  | ---LMYH |  |  |
| Mitogen-activated protein kinase kinase kinase 3 | MAP3K3 | hu | 83.33 | FAQLMY |  | Cytosol |
|  |  | rat | 66.67 | FAQLVY |  |  |
|  |  | ms | 66.67 | FAQLVY |  |  |
| Transmembrane protease serine 4 | TMPRSS4 | hu | 50.00 | CQGDSGGPLMYQ | Cell membrane |  |
|  |  | rat | 41.67 | CQGDSGGPLMYH | Secreted |  |
|  |  | ms | 41.67 | CQGDSGGPLMYH |  |  |

| **CRMKFYSEYC** | | | | | | |
| --- | --- | --- | --- | --- | --- | --- |
| **Protein name** | **Gene name** | **Percent identity** | | **RMKFYSEY** | **Location (extracellular)** | **Location (intracellular)** |
| Cohesin subunit 2 | STAG2 | hu | 57.14 | MKYYNDY |  | Cytoskeleton |
|  |  |  |  | MKMYSD- |  | Cytosol |
|  |  | rat | 57.14 | MKYYNDY |  | Nucleus |
|  |  |  |  | MKMYSD- |  |  |
|  |  | ms | 57.14 | MKYYNDY |  |  |
|  |  |  |  | MKMYSD- |  |  |
| Cohesin subunit 1 | STAG1 | hu | 71.43 | MKYYNDY |  | Cytoskeleton |
|  |  |  |  | MKMYSD- |  | Cytosol |
|  |  | rat | 71.43 | MKYYNDY |  | Nucleus |
|  |  |  |  | MKMYSD- |  |  |
|  |  | ms | 71.43 | MKYYNDY |  |  |
|  |  |  |  | MKMYSD- |  |  |
| Haloacid dehalogenase-like hydrolase domain-containing 5 isoform 2 precursor | HDHD5 | hu | 71.43 | MKLFSEY |  | Mitochondrion |
|  |  | rat | 42.85 | MKLFLQY |  |  |
|  |  | ms | 42.85 | MKLFLQY |  |  |
| Bifunctional arginine demethylase and lysyl-hydroxylase JMJD6 | JMJD6 | hu | 71.43 | MKYYIEY | Cell membrane | Cytosol |
|  |  | rat | 71.43 | MKYYIEY |  | Nucleus |
|  |  | ms | 71.43 | MKYYIEY |  |  |
| DnaJ homolog subfamily C member 8 | DNAJC8 | hu | 83.33 | MTFYSE | Cellular junction | Cytosol |
|  |  | rat | 83.33 | MTFYSE |  | Nucleus |
|  |  | ms | 83.33 | MTFYSE |  |  |
